# SHIV.D Infection Alters Production and Protein Composition of Myeloid-Derived Extracellular Vesicles

**DOI:** 10.3390/ijms27020966

**Published:** 2026-01-18

**Authors:** Rachel M. Podgorski, Amir Yarmahmoodi, Stephen Baak, Rebecca Warfield, Jake A. Robinson, Jennifer Roof, Maurizio Caocci, Hossein Fazelinia, Lynn A. Spruce, Katharine J. Bar, Tricia H. Burdo

**Affiliations:** 1 Center for NeuroVirology and Gene Editing, Department of Microbiology, Immunology, and Inflammation, Lewis Katz School of Medicine, Temple University, Philadelphia, PA 19140, USA; rmpodgorski@gmail.com (R.M.P.); 18baakst@gmail.com (S.B.); rkwarfield95@gmail.com (R.W.); 2 Flow Cytometry Core Facility, Department of Microbiology, Immunology, and Inflammation, Lewis Katz School of Medicine, Temple University, Philadelphia, PA 19140, USA; amir.yarmahmoodi@temple.edu; 3 Department of Medicine, Perelman School of Medicine, University of Pennsylvania, Philadelphia, PA 19104, USA; jake.robinson@pennmedicine.upenn.edu (J.A.R.); bark@pennmedicine.upenn.edu (K.J.B.); 4 Proteomics Core Facility, Children’s Hospital of Pennsylvania, University of Pennsylvania, Philadelphia, PA 19104, USA; roofj@chop.edu (J.R.); fazeliniah@chop.edu (H.F.); spruce@chop.edu (L.A.S.); 5 Department of Medicine, Rutgers Institute of Translational Medicine and Science, Robert Wood Johnson Medical School, Rutgers, The State University of New Jersey, New Brunswick, NJ 08901, USA; mc3044@rbhs.rutgers.edu

**Keywords:** extracellular vesicles, neuroinflammation, SHIV, HIV, viral persistence

## Abstract

Although neurological disease is common in people with human immunodeficiency virus (HIV) (PWH), the contributing factors and underlying inflammatory mechanisms remain challenging to identify. Extracellular vesicles (EVs) constitute a relatively uncharacterized modality of intercellular communication and bioactive cargo transport in the setting of viral infection and pathogenesis. EVs carry inflammatory mediators to areas of the periphery during antiretroviral therapy (ART) suppression but are understudied in the brain. Using a biologically relevant simian–human immunodeficiency chimeric virus with a clade D HIV envelope (SHIV.D)-infected rhesus macaque (RM) model of HIV persistence in the central nervous system (CNS), we investigate circulating EV populations and the protein cargo of myeloid-derived EVs during SHIV infection. Using EV flow cytometry to quantify specific EV subpopulations, we found a significant increase in TMEM119+ microglial EVs and CD171+ neuronal EVs in RM plasma during viremia and ART suppression. Using primary RM monocyte-derived macrophages (MDMs), we determined that MDMs increased EV production after SHIV infection. Whole proteomic analysis of these EVs demonstrated that myeloid EVs isolated from SHIV.D-infected MDMs carried significantly increased levels of neuropathogenic and inflammatory proteins. Altogether, these studies improve our understanding of the contribution of myeloid EVs to neurological disease during SHIV/HIV infection.

## 1. Introduction

Extracellular vesicles (EVs) are membrane-bound nanoparticles released by all cell types [1], broadly classified into exosomes, microvesicles, and apoptotic bodies according to their biogenesis pathways and size profiles [2]. Exosomes are smaller EVs, approximately 50–200 nm, formed from the inward budding of multivesicular bodies from endosomal membranes after fusion with the cells’ plasma membrane. While microvesicles are typically larger EVs, their size can range widely from 50 to 1000 nm [1,2]. Microvesicles are formed by outward budding of the cell’s plasma membrane [2]. Apoptotic bodies, the largest variety at 1000–5000 nm in diameter, are formed by membrane blebbing during programmed cell death [3]. Classical markers of endosome-derived exosomes include tetraspanins CD9, CD81, and CD63, as well as heat shock proteins, flotillin-1 (FLOT1), tumor susceptibility gene 101 (TSG101), and ALG-2 interacting protein X (ALIX) [4,5]. While the biogenesis of microvesicles differs from that of traditional exosomes, they too possess tetraspanins and are enriched for many of the same proteins, such as TSG101, from plasma membrane budding [5,6]. Although apoptotic bodies also form from the plasma membrane, they are significantly larger than exosomes and small microvesicles and generally lack tetraspanins and other classical EV markers [7]. Unlike exosomes and microvesicles, apoptotic bodies contain organelles and other cellular components, making their proteomic profile indistinguishable from cell lysate [6]. Due to their similarities in size, cargo, enriched proteins, tetraspanins, and other membrane markers, it is impossible to differentiate exosomes and small microvesicles in EV preparations [4]. Therefore, it is most accurate to refer to all circulating sub-micron, tetraspanin-positive, membrane-bound nanoparticles as EVs.

As membrane protein-expressing vesicles, EVs are important players in intercellular communication, containing proteins, lipids, and nucleic acids (DNA fragments, messenger RNA, microRNA, and non-coding RNA) [4,5,8]. EVs are released into the extracellular space, are detected in all bodily fluids [8], and even cross the blood–brain barrier (BBB) [9]. Intercellular signaling through EVs can occur through multiple biological processes. EVs can influence target cells by interacting with their cell surface without being internalized [10]. Recipient cells can take up heterogeneous EVs through a variety of mechanisms, including macropinocytosis, phagocytosis, membrane fusion, lipid raft-mediated endocytosis, and receptor–ligand-mediated endocytosis [11]. Following uptake, EVs co-localize with receptor cell endosomes and lysosomes, and most likely release their cargo into the cytosol through EV membrane degradation [11,12]. Some research demonstrates that EVs can interact with specific cell types. One study exposing urologic cancer cell-derived EVs to cancerous and noncancerous cells of different tissue types resulted in more efficient EV uptake by cancerous cells [13]. Another study demonstrated that EVs produced by cortical neurons after synapse activation were endocytosed exclusively by neurons and did not interact with glial cells [14]. Due to their ability to retain membrane proteins and cargo from their cell of origin, dissemination throughout the body, and specific nature of intercellular signaling, EVs are a valuable source of cellular information as biomarkers or effectors of physiological and pathological processes in vivo.

The roles of EVs in the pathogenesis of central nervous system (CNS) disease have been of particular interest due to the persistent and complex causative nature of neurodegeneration and neuroinflammation. Brain-derived EVs can be found in the blood and cerebral spinal fluid (CSF), providing a window into ongoing neuropathological processes in patients and animal models of diseases over time. Early exosome research found that pathogenic misfolded prion proteins can be packaged into EVs, and these EVs can propagate prion disease in a naïve animal host [15]. Soon after, it was discovered that EVs serve as both biomarkers of early Alzheimer’s disease as well as mediators of pathogenesis [16]. EVs containing amyloid-β, amyloid precursor protein, and tau transport these proteins across the brain and initiate new accumulations [16,17,18].

There is growing interest in exploring the functions of EVs in human immunodeficiency virus (HIV) pathogenesis. EVs have a unique relationship with viral infections, as viruses can co-opt EV biogenesis pathways for virion production in the host cell, mimicking intra- and intercellular trafficking and communication [19,20]. During HIV infection, the virus hijacks the host’s exosome production machinery, the Endosomal Sorting Complex Required for Transport (ESCRT) pathway, for virion budding [20,21]. The functions and utility of EVs are of particular interest in the neurological pathogenesis of HIV. Some EVs have been identified as drivers of neuropathogenesis during HIV infection. Astrocyte-derived EVs (ADEVs) containing HIV nef are taken up by neurons in a co-culture model, decreasing neuronal action potentials [22]. Mice receiving ADEV from cells stimulated with HIV tat exhibit behavioral changes and synaptodegeneration [23]. EVs from HIV-infected macrophages transport viral particles and inflammatory cytokines to nearby uninfected cells [24,25]. EVs from infected cells can carry functionally active kinases, which interfere with the cell cycle dynamics of bystander cells [26]. Proteomic profiling of EV cargo from ART-suppressed people with HIV (PWH) reveals an upregulation of inflammatory interferon-gamma and interleukin 1-α [27]. Targeted protein analysis of plasma neuron-derived EV cargo demonstrates differential expression of several proteins between PWH with and without cognitive impairment [28,29,30]. Genes that are upregulated in PWH experiencing cognitive impairment are of note as potential biomarkers. A mass spectrometry study comparing CSF EV cargo from PWH with and without cognitive impairment found higher abundance of genes associated with stress response and inflammation by gene ontology in PWH experiencing neurological symptoms [31]. In addition to viral and host proteins, EVs from HIV-infected cells contain significantly different microRNA (miRNA) profiles [25]. EVs isolated from HIV-infected macrophages in vitro have several differentially upregulated miRNAs compared to their uninfected counterparts, including pro-inflammatory miR-29a [32]. In a study profiling EV-associated miRNAs from ART-naïve PWH, two miRNAs associated with inflammatory pathogenesis were identified [33]. A recent study of 84 PWH on virally suppressed ART found HIV RNA in EVs derived from CSF and serum EVs. The EVs were more abundant in CSF and correlated with neurocognitive dysfunction [34]. Lastly, multiple studies have demonstrated an increase in cellular TNF-α production in vitro after treatment with EVs from HIV-infected cells [35,36].

HIV-1 persistence in the CNS is driven by infection of myeloid lineage cells [37]. HIV seeding of the CNS is mediated by the transmigration of infected macrophages, CD14+/CD16+ monocytes, and CD4+ T lymphocytes across the blood–brain barrier [37,38,39]. Resident CNS macrophages and microglia are susceptible to HIV infection and harbor proviral DNA and low levels of replicating viral RNA during ART-induced plasma viral suppression [38,39]. These long-lived myeloid cells form the viral reservoir in the CNS, which persists during ART suppression and can rebound into active viral replication if treatment is interrupted [40].

There is a strong connection between EVs and inflammation in the context of HIV infection, but cell-specific EV production and host protein cargo over the course of a non-human primate (NHP) model of HIV infection have not been explored. Chimeric simian–human immunodeficiency viruses (SHIVs) have several properties that are advantageous for use in modeling CNS HIV infection in NHP. SHIVs are engineered viruses in which an HIV gene replaces an equivalent SIV gene. SHIV.D.191859 (SHIV.D) is a chimera of a transmitted/founder (TF) SIV backbone that encodes an HIV-1 clade D envelope, which is C-C chemokine receptor type 5 (CCR5)-tropic, with the ability to replicate in both CD4+ T cells and macrophages [41]. SHIV.D infection of RM recapitulates many features of HIV-1 pathogenesis, including mucosal and intravenous transmission, consistent viral kinetics, and CD4 depletion over time [42]. Historically, infection with clade D HIV is associated with more severe neuropathogenesis and higher prevalence of HIV-associated neurocognitive decline than clades B, C, and A [43]. In a study of SHIV.D persistence in the RM brain during ART suppression, quantitative immunohistochemistry and DNA/RNAscope in situ hybridization revealed that myeloid-mediated inflammation, viral replication, and proviral DNA persist in the brains of RMs after six months of ART suppression [44]. The desirable viral kinetics, myeloid tropism, and CNS persistence of SHIV.D provide a valuable opportunity to study EVs involved in HIV neuropathogenesis in an RM model. Here, we aim to phenotype circulating EV subpopulations and assess their dynamics during SHIV.D infection and ART-mediated viral suppression in rhesus macaques (RMs) to identify potential neuroHIV-related EV signatures. Additionally, we aim to characterize EVs derived from SHIV.D-infected rhesus monocyte-derived macrophages (MDMs), including their cargo, and compare them to EVs from uninfected controls to understand how myeloid-driven EVs may contribute to brain inflammation and neuropathogenesis.

## 2. Results

### 2.1. Macrophage-Derived Extracellular Vesicles (MEVs) Are Differentially Produced During SHIV.D Infection

**Figure 1:**
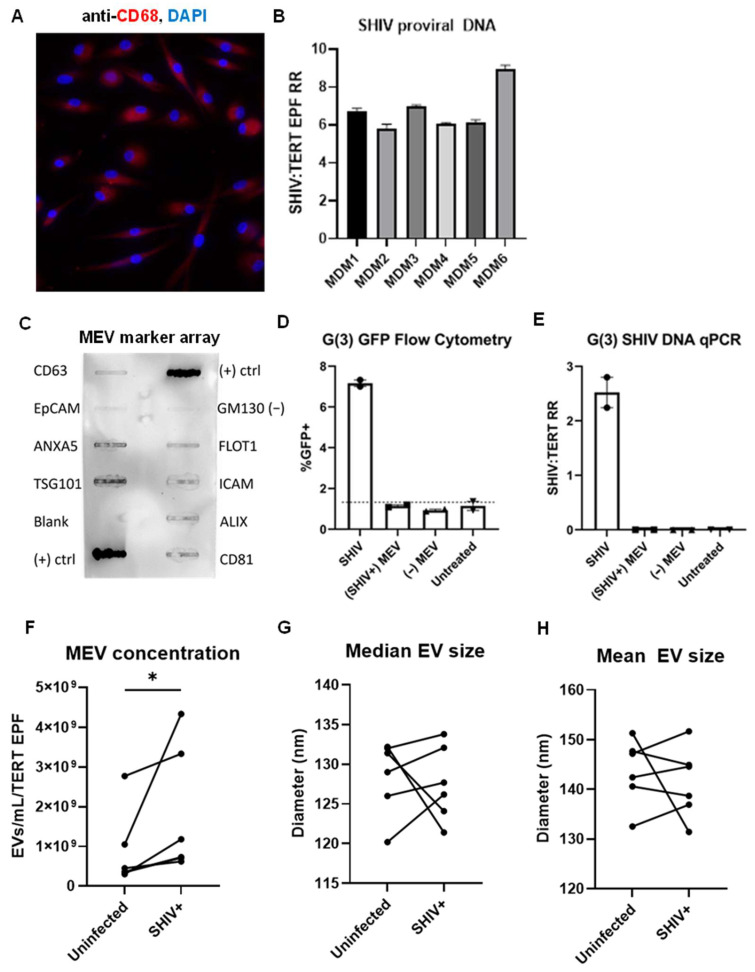
Characterization of macrophage EVs (MEVs). (**A**) Primary RM MDM expresses the macrophage marker CD68 (red). Magnification 40× (**B**) MDMs were infected with SHIV.D as they contain proviral DNA. (**C**) MEVs express EV markers CD63, ANXA5, TSG101, FLOT1, ICAM, ALIX, and CD81. (**D**,**E**) EVs isolated from SHIV.D+ MDMs are not infectious. Flow cytometry for GFP, indicating SHIV infection (**D**), demonstrated no GFP expression above baseline untreated expression (dotted horizontal line) in Ghost(3) (G(3)) reporter cells treated with SHIV+ MEV. Proviral SHIV DNA was absent in G(3) cells treated with SHIV+ MEV (**E**). (**F**–**H**) Each line represents MDMs from one RM uninfected compared to the same RM infected. MDMs from six different RMs were used as biological replicates. (**F**) MEV concentration normalized to RM TERT endpoint fluorescence (EPF) is significantly increased with SHIV.D infection (Wilcoxon paired *t*-test, * *p* = 0.0313, N = 6). The median (**G**) and mean (**H**) EV size are shown.

Following differentiation, MDMs express tissue macrophage marker CD68 and display typical elongated M2 morphology [45] (Figure 1A). MDM infection with SHIV.D resulted in the production of viral RNA in the cell culture supernatant, as well as the presence of proviral DNA in the cells (Figure 1B). Pooled MEVs express markers CD63, ANXA5, TSG101, FLOT1, ICAM, ALIX, and CD81 at higher intensity than cellular protein control GM130 (Figure 1C). Reporter cell treatment with MEV from SHIV−/+ MDM demonstrated that EVs isolated from SHIV-infected cells do not contain competent virions and are not infectious themselves. qPCR did not detect SHIV proviral in cells treated with SHIV+ MEV, and reporter cells did not yield GFP expression above baseline (Figure 1D,E). Nanoparticle tracking analysis (NTA) revealed nanoparticles of appropriate size (Figure 1G,H). MEV concentration determined by NTA normalized to RM tert endpoint fluorescence (EPF) demonstrated a significant increase in MEV production in the SHIV+ MDM (*p* = 0.03, n = 6) (Figure 1F). In uninfected MDMs, the average (arithmetic mean) EV concentration relative to RM tert expression was 8.77 × 10^8^ EVs/mL. From SHIV.D+ RM, the average EV concentration was 1.82 × 10^9^ EVs/mL. There was no change in EV size between SHIV.D−/+ groups (Figure 1G,H). The average median MEV diameter was 128.5 nm from uninfected MDMs and 127.6 nm from SHIV.D+ MDMs. The average mean MEV diameter was 143.6 nm in uninfected cells and 141.4 nm in viremic cells.

### 2.2. Characterization of RM Plasma EVs

**Figure 2:**
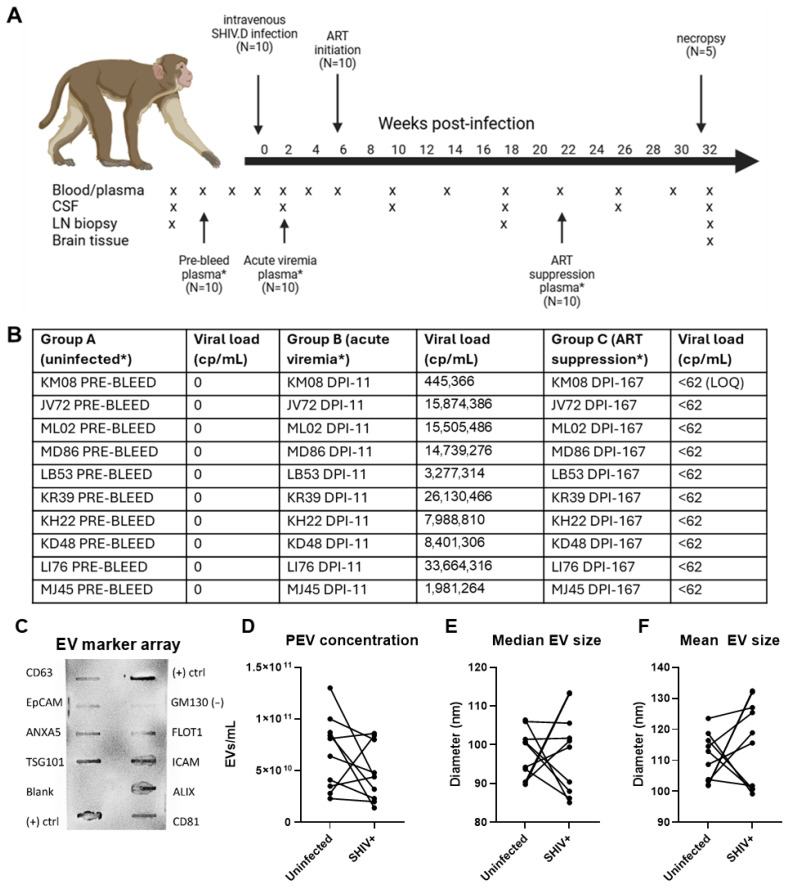
Characterization of RM plasma EVs (PEVs). Ten RMs were infected with high-titer SHIV.D intravenously. (**A**) Blood, CSF, LN, and brain tissue samples were taken at the timepoints identified. Pre-bleed (uninfected) plasma, acute viremia plasma, and ART-suppression plasma from identified timepoints (*) were used for nanoparticle tracking analysis (NTA) and flow cytometry. (**B**) Ten RM plasma samples used for EV isolation and analysis, and their viral loads (copies/mL). (**C**) PEV express EV markers CD63, ANXA5, TSG101, FLOT1, ICAM, ALIX, and CD81. PEV concentration (**D**), median (**E**), or mean (**F**) size are shown in matching uninfected or SHIV.D+ viremic plasma in ten RMs. Antiretroviral therapy (ART), day post infection (DPI), limit of quantification (LOQ), lymph node (LN). * indicates corresponding timepoints for blood draws.

EVs were isolated from plasma samples from RMs before infection with SHIV.D, during viremia, and during ART-induced viral suppression (Figure 2A,B). Isolation of RM plasma EVs (PEVs) was validated by ZetaView NTA and EV marker antibody array (Figure 2C–F). Pooled RM PEVs express markers CD63, EpCAM, ANXA5, TSG101, FLOT1, ICAM, ALIX, and CD81 at higher intensity than cellular protein control GM130 (Figure 2C). NTA revealed the presence of appropriately sized nanoparticles. Using NTA, total PEVs showed no significant difference in concentration or size between RM plasma before infection and SHIV.D viremia at day post infection (DPI) 11 (Figure 2D). The mean PEV concentrations were 6.73 × 10^10^ EVs/mL plasma in uninfected RMs and 4.75 × 10^10^ EVs/mL plasma in infected samples. The average (arithmetic mean) of median PEV diameter was 97.37 nm in uninfected RMs and 98.40 nm in viremic RMs. The average mean PEV diameter was 110.6 nm in uninfected RMs and 115.4 nm in viremic RMs (Figure 2E,F).

### 2.3. EV Flow Cytometry Workflow, Validation, and Gating Strategies

To analyze changes in specific cell-derived EV populations in the plasma over time, we developed a novel nanoscale multiplex conventional flow cytometry method capable of analyzing circulating EVs by markers of cellular origin (Figure S1). To calibrate, validate, and optimize this technique, several controls were used [46,47]. FITC-conjugated sub-micron particle size reference beads (100 nm, 200 nm, 500 nm, and 1000 nm; Thermo Fisher Scientific, Waltham, MA, USA) were used to approximate EV side scatter (SSC) and optimize flow rate and detection threshold (Figure S2). Reference bead size SSC can only be approximated, as polystyrene beads have different refractivity and complexity than EVs. Unstained EVs were used to generate EV forward scatter (FSC) and SSC gates, and a suspension of the antibody cocktail in PBS 1% PFA was run to ensure antibody aggregates were not creating false positive events (Figure S3). Additionally, a preparation of PEVs was treated with Triton X-100 detergent to lyse EV membranes. When acquired for 5 min, the Triton X-100-treated sample demonstrated a 70% reduction in events compared to an untreated sample, which is consistent with the literature [48,49]. Positive CD81+ gating was determined by comparing fluorescent intensity between the fluorescence minus one (FMO) control and the compensation control (Figure S4). For each parameter, the mean fluorescence intensity (MFI) of each channel was used to determine the relative abundance of CD81+ EVs expressing each marker. Each sample used for FMO and compensation controls was obtained from the same RM at the same timepoint to avoid the natural variation in EV profiles that occurs between animals and over time. Single color compensation controls were used to optimize voltages and set compensation matrix parameters (Table S1). FMO controls, which contain each antibody in the panel except one, were used in conjunction with compensation controls to determine positive and negative gating. For each compensation control, 5000 events were recorded. For each FMO control, 10,000 events were recorded. Two panels were developed to minimize signal spillover and increase detection of rare events (Table S2). Each panel contained three markers of cellular origin and one EV marker (tetraspanin CD81). RM plasma EVs isolated as described in Figure S1 were used for each single-color compensation control and FMO control.

### 2.4. Microglia- and Neuron-Derived EVs Are Significantly Increased During Viremia and ART Suppression

**Figure 3:**
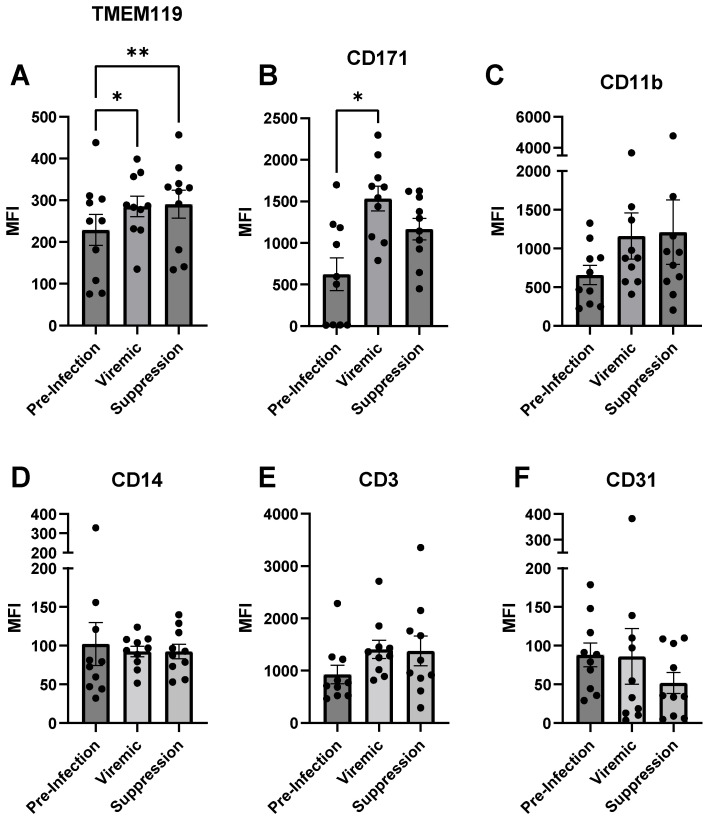
Neuronal and microglial CD81+ EVs are increased in plasma during viremia and ART suppression in ten non-human primates. The mean fluorescence intensity (MFI) of (**A**) TMEM119+ microglial EVs, (**B**) CD171+ neuronal EVs, (**C**) CD11b+ macrophage EVs, (**D**) CD14+ monocyte EVs, (**E**) CD3+ T cell EVs, and (**F**) CD31+ endothelial EVs was measured via flow cytometry in CD81+ EVs of plasma from 10 rhesus macaques during pre-infection, viremia, or viral suppression. (**A**) TMEM119+ microglial EVs are significantly increased in RM plasma during viremia and ART suppression, and (**B**) CD171+ neuronal EVs are significantly increased in the plasma during viremia. The means and standard error of the mean (SEM) are shown. Repeated measures one-way ANOVA was performed, and if significant *p* < 0.05, it is followed by post hoc paired *t*-tests. * = *p* < 0.05, ** = *p* < 0.01.

Plasma samples from RMs before infection with SHIV.D, during viremia, and during ART-induced viral suppression (Figure 2A,B) were prepared for each flow cytometry panel, and sample acquisition and analyses were performed as described above. By repeated measures one-way ANOVA, we found that CD171+ neuron-derived EVs (*p* = 0.01) and TMEM119 microglia-derived EVs (*p* = 0.008) were significantly different between the three groups (Figure 3). Post hoc paired *t*-tests comparing TMEM119+ microglial EV MFI also demonstrated a significant increase in viremic RMs (*p* < 0.05, mean MFI = 285.6) and ART-suppressed RMs (*p* = 0.004, mean MFI = 290.8) compared to pre-infection RMs (mean MFI = 229.0) (Figure 3A). Post hoc paired *t*-tests revealed that neuronal CD171+ EVs are significantly increased in both viremic RMs (*p* = 0.03, mean MFI = 1535) and ART-suppressed RMs (*p* = 0.04, mean MFI = 1167) compared to those of RMs before infection (mean MFI = 624.5) (Figure 3B). There were no differences between TMEM119+ or CD171+ EVs between viremic and ART suppression, so even with controlled ART, these EV populations were elevated. While the average CD171 MFI between groups was highest in viremic RMs, the average TMEM119 MFI was highest in ART-suppressed RMs. Though not statistically significant, CD11b pan-myeloid/macrophage-derived EVs and CD3 T cell-derived EVs also showed an increase in average MFI during viremia and ART suppression compared to their pre-infection control samples.

### 2.5. MEV Protein Cargo Is Significantly Changed with SHIV.D Infection

**Figure 4:**
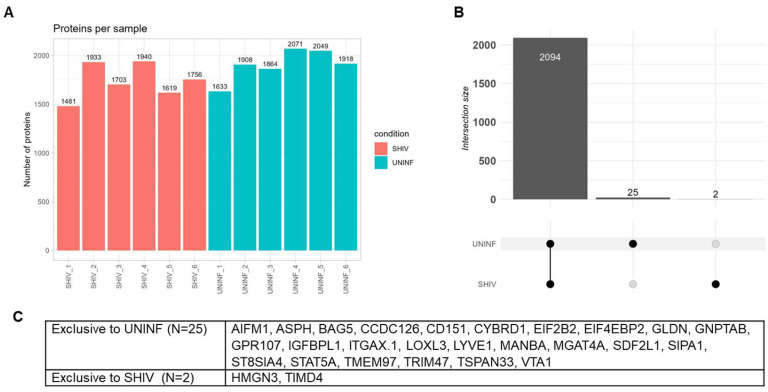
Proteins per sample and condition, exclusive proteins. (**A**) Number of proteins detected in each sample after removal of proteins that were found in fewer than 4/6 samples per condition. (**B**) Number of proteins detected in 4/6 samples in both conditions (2094), UNINF only (25), and SHIV only (2). (**C**) UniProt GeneID proteins exclusive to each condition.

Primary MDMs were differentiated from the PBMCs of six healthy RMs, and half of the MDMs from each RM were infected with SHIV.D at an MOI of 1. Pooled cell culture supernatant from DPI 2–8 was harvested at the same time from SHIV.D−/+ MDM and used for EV isolation and proteomic analysis. In the analysis, proteins that were detected in at least four of the six replicates of at least one condition were included. Quantification of filtered results identified an average (arithmetic mean) of 1739 proteins per sample in the SHIV.D+ condition and 1907 proteins per sample in the uninfected condition (Figure 4A). A total of 2094 of the total proteins identified and qualified for analysis were present in both conditions. Twenty-five proteins were found only in the uninfected cohort, and two proteins were exclusive to the SHIV.D+ cohort (Figure 4B,C).

**Figure 5:**
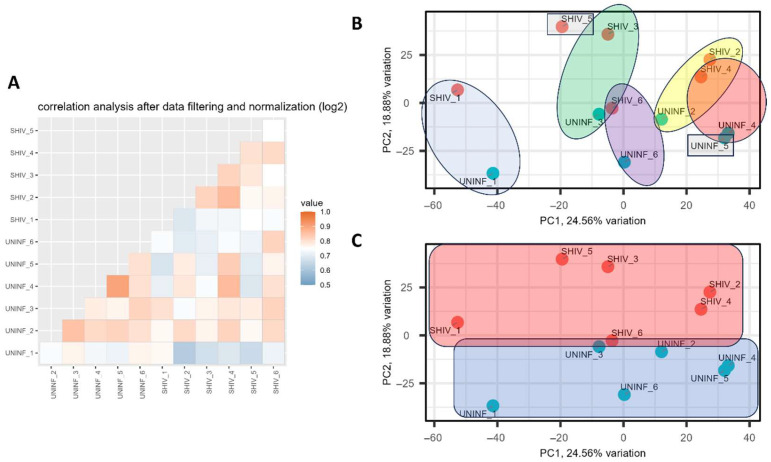
Correlation analysis and principal component analysis (PCA). (**A**) Correlation between samples after data filtering and normalization. Orange tones indicate a higher level of correlation than blue tones. (**B**) Two-dimensional PCA plot of the first two principal components with ellipses indicating clustering between paired samples from the same RM donor. (**C**) PCA plot with rectangles indicating clustering between samples of the same condition. SHIV+ samples are highlighted in red, and uninfected samples are highlighted in blue.

Data were prepared by log2 transformation, filtering, normalization, and data imputation for correlation analysis, principal component analysis (PCA), differential abundance analysis, overrepresentation analysis, and gene set enrichment analysis (GSEA). Correlation analysis generally demonstrated stronger correlations between samples of the same condition as well as between SHIV.D−/+ samples from the same donor RM (Figure 5A). PCA compares the variances in peptide makeup of each sample in a two-dimensional plot of the two most significant principal components. PCA shows that, generally, samples from the same RM donor cluster together (Figure 5B), as do samples from the same condition (Figure 5C).

**Figure 6:**
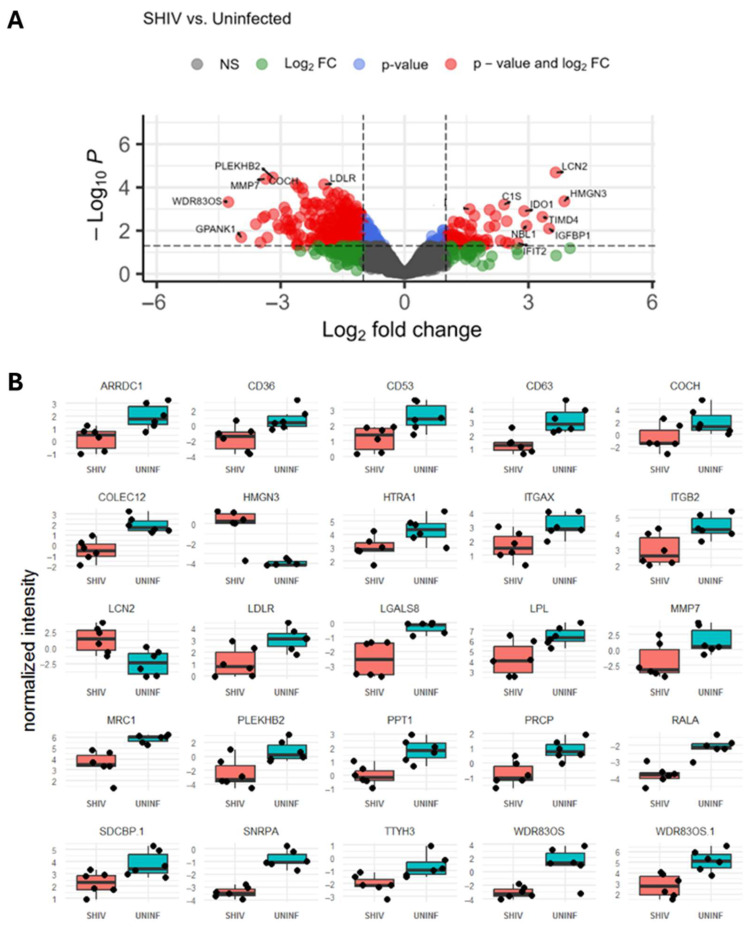
Volcano plot and normalized intensities of differentially abundant proteins between conditions. (**A**) Differentially abundant proteins are graphed on a volcano plot by log2 fold change (FC) of SHIV vs. uninfected conditions. Proteins that are significant by *p*-value (*p* < 0.05) and log2 FC are denoted in red. Green: log2 FC only; blue: *p*-value only; gray: not significant (ns). (**B**) Normalized intensity of each significantly differentially abundant protein in each sample was plotted to compare affected proteins in UNINF (blue) and SHIV (red) conditions (Table S4).

Differential abundance analysis was performed as described, comparing the SHIV condition to the uninfected condition (UNINF). The most significantly differentially abundant proteins are described (Table S4) and plotted by normalized intensity in each condition (Figure 6B). Only 2 of the 25 most significantly differentially abundant proteins were upregulated in the SHIV condition: lipocalin-2 (LCN2) and high mobility group nucleosome-binding domain-containing protein 3 (HMGN3). A volcano plot was generated to visualize differentially abundant proteins between groups by log2 fold change and *p*-value (Figure 6A). With LCN2 and HMGN3, other significantly overabundant proteins in SHIV EVs include T cell immunoglobulin and mucin domain containing 4 (TIMD4), neuroblastoma suppressor of tumorigenicity 1 (NBL1), insulin-like growth factor binding protein 1 (IGFB1), and indoleamine 2,3-dioxygenase 1 (IDO1). Proteins with a significant negative fold change in a SHIV vs. UNINF comparison are differentially overabundant in the UNINF EVs. Differential abundance analysis identified a greater number of overrepresented proteins in the UNINF condition overall. Lastly before pathway analysis, 437 significant proteins were organized in a heatmap, where unbiased relational protein clustering was observed between SHIV and UNINF groups (Figure S5).

### 2.6. Gene Ontology Pathway and Component Associations of EV Proteins

**Figure 7:**
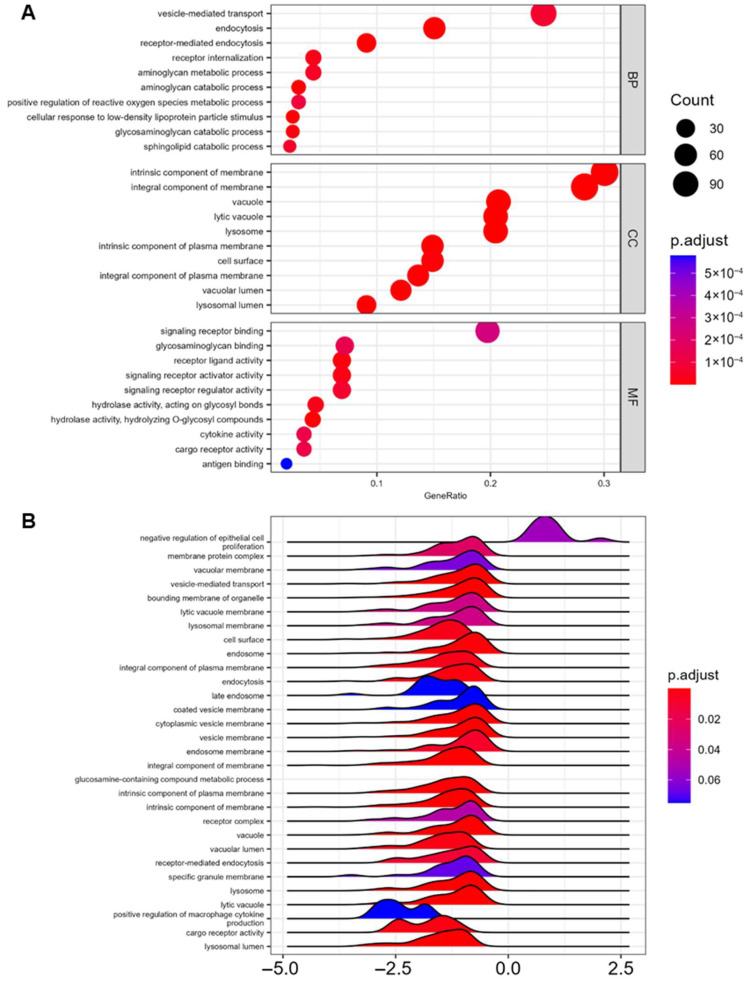
Gene ontology (GO) and gene set enrichment analysis (GSEA). (**A**) Biological processes (BPs), cellular components (CCs), and molecular functions (MFs) with associated proteins that were overrepresented in the total data set are identified. GeneRatio: percentage of differentially abundant proteins in each GO term. (**B**) GSEA was performed on GO pathways that differed between conditions using human GO terms on the total protein data set without *p*-value restrictions. Fold change in SHIV vs. UNINF is indicated on the *x*-axis.

The proteins associated with specific biological processes (BPs), cellular components (CCs), and molecular functions (MFs) were identified using a gene ontology database. A *p*-value cutoff of <0.0005 was used to generate a list of genes of interest. Gene ontology analysis identified the BPs, CCs, and MFs associated with the most significantly differentially abundant proteins between conditions (Figure 7A). Of the BP pathways, proteins associated with vesicle-mediated transport, endocytosis, receptor-mediated endocytosis, and receptor internalization were the most abundant and most significantly different. Proteins associated with the positive regulation of reactive oxygen species (ROS) were also represented. Among the CCs, membrane, vacuole, and lysosome components were overrepresented. Among the MFs, signaling receptor binding was the most abundant pathway.

Other signaling and receptor activities, cytokine activity, and cargo receptor activity were also MF pathways of proteins differentially abundant among MEVs. Proteins associated with glycosaminoglycan catabolic processes and glycosyl bond hydrolysis pathways were also overrepresented among the significant data (Figure 7A). An enrichment map network was generated to visualize the fold change in proteins associated with the most significantly overrepresented gene ontology (GO) pathways (Figure S6). Several proteins associated with glycosaminoglycan and aminoglycan catabolic processes were downregulated in SHIV EVs compared to the UNINF condition. The pathway with the most differentially abundant proteins was endocytosis. Of the relevant proteins with the most significant negative fold change from UNINF to SHIV, several are associated with positive immune regulation and cell proliferation. The most significantly overexpressed protein in SHIV EVs associated with the endocytosis GO pathway is TIMD4, which was also one of the most significantly overrepresented proteins in the SHIV condition overall (Figure S6). Lastly, GSEA using human GO terms was conducted using the entire data set and plotted with the fold change in each pathway comparing the SHIV condition to the UNINF (Figure 7B). GSEA revealed that GO pathways of the entire data set (unbiased to proteins with differential abundance) are primarily downregulated in SHIV EVs compared to UNINF. The pathways with the most significant negative fold change are largely associated with vesicles, vacuoles, and plasma membranes. The only GO pathway with a significant positive fold change in the SHIV condition was negative regulation of epithelial cell proliferation (Figure 7B).

## 3. Discussion

The increase in MEV production after SHIV.D infection in vitro necessitated further study into EV subset dynamics and cargo in a SHIV.D-infected NHP model. Our significant findings of sustained increases in circulating neuron and microglia-derived EVs during ART suppression are the first of their kind due to the distinctive ability to investigate EV production before infection and over time in an NHP model of HIV. These findings reinforce the importance of EVs in HIV infection but raise further questions about their roles. From these data alone, it is unknown if the increase in circulating microglial and neuronal EVs is due to an increase in their release into the periphery or a result of BBB injury and increased permeability. Nevertheless, each possibility demonstrates that EVs act as mediators of disease via cargo release or cell signaling, or as biomarkers of ongoing neuropathogenesis. Previous research has compared CD171+ neuron-derived EVs from PWH with and without cognitive impairments and found no change in the number of EVs between these groups [50]. However, the cargo of neuronal EVs has been demonstrated to contain proteins associated with neurodegeneration, inflammation, and neuronal injury [28,29,50]. Our matched analysis of cell-specific EVs throughout the course of infection and treatment in the same RM is a more robust technique than unpaired analysis, but further research is necessary to determine if the sustained increase in neuronal and microglial EVs during ART suppression will be recapitulated in a larger study size or in PWH.

Unlike CD171/L1CAM+ EVs, TMEM119+ microglial-derived EVs are infrequently studied, and their relationship to HIV infection is novel. The significant increase in TMEM119+ EVs during acute infection and additional increase during ART suppression is a novel finding and indicates that microglial-derived EVs should be further investigated in the contexts of HIV and chronic neurological dysfunction. A recent study reported that plasma EVs from PWH were enriched with bone and kidney markers based on spectral flow analyses, and levels were higher in PWH with cognitive impairment [51]. Consistent with our findings, the study observed no difference in overall plasma EV counts across neurological status in PWH, but they did not examine EV cargo or neuronal markers [51].

Our study applies conventional flow cytometer/cell sorter for bead-free multiplex analysis of EVs, enabling simultaneous quantification of multiple EV surface antigens from a single plasma sample. EV-associated tetraspanins CD81 and CD63 are both readily detectable, but CD81, more prevalent in RM plasma EVs and expressed on a broader range of brain- and immune cell-derived EVs, was used as an internal control in both panels [52,53]. Markers of cellular origin were observed at expected frequencies, and rare populations, such as CD171/L1CAM+ neuron-derived EVs, were also detectable [54]. The absence of detectable CD171+ neuron-derived EVs in four of ten uninfected RMs suggests notable inter-individual variability in baseline neuronal EV release.

This technique was designed to quantify plasma EV subpopulations, focusing on brain- and immune-derived EVs to gain insight into otherwise difficult-to-assess disease processes. This protocol improves upon existing methods by combining plasma separation and defibrination, polymer-based EV precipitation, and nanoparticle filtration, enabling enrichment and purification of the EV fraction. This protocol was developed in accordance with guidelines recommended by both the minimum information for studies of EVs (MISEV) framework and the minimum information about a flow cytometry experiment standard in an EV-specific framework (MIFlowCyt-EV) [46,47]. The primary limitation is the use of CD81 backbone EV marker, as it may exclude some tetraspanin-negative EVs [52,53]. This approach excludes lipoprotein contaminants, which lack tetraspanins [55]. Overall, circulating brain-derived EV populations show promise as biomarkers or mediators of sustained neuropathogenesis in treated HIV infection.

Our findings that myeloid-derived EVs are increased in the plasma of RMs during SHIV.D infection and ART suppression necessitated further study. Using an in vitro primary RM myeloid cell model and nanoparticle tracking analysis, we demonstrate that myeloid cells increase EV production after SHIV.D infection. Because SHIV.D forms a reservoir in myeloid cells in the CNS and is associated with myeloid-mediated neuropathogenesis, we hypothesized that EVs produced by SHIV-infected myeloid cells may carry protein mediators of pathogenesis as cargo. Future studies should explore proteomic profiling of TMEM119+ microglial EV subpopulations in addition to SHIV.D infected monocyte-derived macrophage EVs.

Bioinformatics analysis of mass spectrometry proteomics data revealed valuable information about the proteomic makeup of MEVs, as well as key differences between the proteins found in EVs derived from uninfected and SHIV-infected myeloid cells. First, data filtering and QC/QA demonstrated high-quality protein samples. Correlation and principal component analysis revealed appropriate clustering between conditions and samples from the same RM donor. Differential abundance analysis highlighted the top 25 most significantly differentially abundant proteins between the two groups. Of those 25, only 2 were increased in the SHIV+ EVs (HMGN3 and LCN2). The most differentially abundant proteins in the SHIV condition with the highest fold change were TIMD4, HMGN3, LCN2, NBL1, IGFB1, and IDO1. Nearly all these proteins have demonstrated neuropathogenic effects or roles in HIV pathogenesis. TIM proteins, including TIMD4, promote HIV entry into the host cell [56,57]. One study revealed that TIMD4+ EVs promote HIV entry into host cells and blocking with anti-TIMD4 antibodies significantly impeded HIV infection [56]. There is no known connection between HMGN3 and HIV or neuropathogenesis, but one report connected elevated neuronal EV-associated levels of a different high mobility group protein, HMGB1, to neuropsychologically impaired PWH [30]. LCN2 is a known mediator of neurotoxicity and neuroinflammation [58,59]. A recent report found that LCN2 drives neuronal injury and behavior deficits in a humanized mouse model of HIV [60]. Increased levels of plasma IGFBP1 are associated with greater levels of inflammation and more severe disease in PWH [61]. IDO1 is a known mediator of HIV neuropathogenesis [62], with plasma IDO1 associated with CNS viral reservoirs [63]. These significantly overabundant pathologic proteins in SHIV+ MEVs give insight into potential mechanisms of MEV-mediated neuroinflammation and CNS pathology.

Interestingly, matrix metalloproteinase 7 (MMP7) was downregulated in SHIV EVs compared to UNINF EVs. In general, proteins in the MMP family are associated with neuronal injury in HIV, but MMP7 inversely correlates with viral load [64]. Most GO pathways enriched in the uninfected group were related to vesicle formation, structure, release, and uptake, suggesting that SHIV/HIV-infected EVs may differ functionally from those of healthy cells. In the SHIV-infected cohort, only negative regulation of epithelial cell proliferation was enriched, a novel finding requiring validation. Other uninfected-enriched pathways included glycosaminoglycan binding and catabolism, highlighting a potential link between SHIV+ EVs and increased myeloid cell infection or pathogenesis.

This study has limitations. First, low total protein in MEV samples likely excluded some proteins from bioinformatics analysis due to filtering criteria (detection in 4/6 samples), contributing to the overrepresentation of proteins in the uninfected EVs. There are also inherent limitations to data imputation, which could influence observed fold changes. Finally, EV sample contamination with co-precipitated lipoproteins is a common obstacle that is difficult to avoid in EV isolation from cultured cells and may account for enrichment of proteins linked to the cellular response to lipoprotein stimulus [55].

Overall, these findings demonstrate that EVs produced by SHIV+ myeloid cells are increased in production and carry protein mediators of neuropathogenesis and neuronal injury. These data support the hypothesis that MEVs perpetuate neuropathogenesis during SHIV/HIV infection and indicate potential mechanisms of persistent neuropathogenesis.

## 4. Materials and Methods

### 4.1. Primary Macrophage Culture and SHIV.D Infection

SHIV.D.191859 was cloned, and viral stock was generated as previously described [42,65,66,67]. Infectivity of the viral stock was calculated by serial stock dilution and infection of Ghost(3) CCR5+ Cells (Hi-5, ARP-3944; a kind gift from Dr. Peter Gaskill of Drexel University College of Medicine with the original stock obtained through the NIH HIV Reagent Program, Division of AIDS, NIAID, NIH), which express GFP upon HIV/SIV-Tat activation of transcription, followed by GFP flow cytometry using Guava easyCyte [68]. RM peripheral blood mononuclear cells (PBMCs) were isolated from EDTA whole blood using a Ficoll (Cytiva, Marlborough, MA, USA) gradient and plated in RPMI medium (ThermoFisher Scientific, Waltham, MA, USA) + 10% exosome-depleted FBS (ThermoFisher Scientific, Waltham, MA, USA) + gentamicin (Corning, Corning, NY, USA) across 6-well plates at a density of 1 million cells/mL. After 24 h in culture, differentiation into monocyte-derived macrophages (MDM) was stimulated with 50 ng/mL recombinant human macrophage colony-stimulating factor (M-CSF (R&D Systems, Minneapolis, MN, USA)). Half media changes with M-CSF were performed on days 4 and 7 after plating. On day 8, cells were washed 2× with PBS (Corning, Corning, NY, USA), and adherent macrophages were infected with SHIV.D.191859 viral stock at an MOI of 0.1–1.0 for 24 h. Following 24 h of incubation with the virus, the infection media was removed, the cells were washed 2× with PBS, and 2 mL of fresh working media (RPMI with 10% exosome-depleted FBS, gentamicin, and M-CSF) was added per well. Working media changes were performed every 24–48 h after initial infection. Cell culture supernatant from each media change was centrifuged for 5 min at 1500 rpm to remove cells and cellular debris, then stored at −80 °C for downstream EV or vRNA isolation.

### 4.2. Immunocytochemistry

To validate differentiation of primary PBMC into mature myeloid cells, immunocytochemistry (ICC) for pan-macrophage marker CD68 was performed. Primary MDMs were fixed in-well with 4% PFA in PBS for 30 min at RT, then washed 3× with PBS. Following fixation, cells were permeabilized with PBST (0.1% Tween-20 (ThermoFisher Scientific, Waltham, MA, USA) + 0.1% Triton-X100 (Acros Organics, Geel, Belgium)) for 15 min, then washed 3 × 2 min with PBST. Blocking was performed with PBST + 2% BSA (ThermoFisher Scientific, Waltham, MA, USA) + 5% FBS (ThermoFisher Scientific, Waltham, MA, USA) for 30 min. Cells were incubated with anti-CD68 primary antibody (M0814, Dako/Agilent Technologies, Carpinteria, CA, USA, 1:50) or mouse IgG negative isotype control (Dako/Agilent Technologies, Carpinteria, CA, USA, 1:50) diluted in blocking solution overnight at 4 °C. Cells were then washed 3 × 5 min with PBST and incubated with anti-mouse secondary antibody (Invitrogen A32744, Carlsbad, CA, USA, 1:1000) for one hour at RT. Following secondary incubation, cells were washed 3 × 5 min in PBST, then stained with DAPI (Akoya Biosciences, Marlborough, MA, USA, 1 drop:1 mL PBST). Residual antibody, tween, and salts were removed with 2 × 5 min PBS wash followed by 2 × 5 min H_2_O wash. Cover slips were fixed to plates using ProLong Gold Antifade Mountant (Thermo Fisher Scientific, Waltham, MA, USA) for storage at 4 °C, and cells were imaged using a Keyence BZ-X700 microscope (Keyence, Osaka, Japan).

### 4.3. qPCR and RT-qPCR

Productive SHIV.D infection of primary RM MDMs in vitro was validated by quantitative polymerase chain reaction (qPCR) of SHIV.D proviral DNA and reverse-transcription qPCR (RT-qPCR) of viral RNA isolated from cell culture supernatant. Double-stranded DNA was isolated from pelleted MDMs using a DNA miniprep kit (Zymo Research, Irvine, CA, USA) and quantified using Qubit dsDNA BR Assay (Invitrogen, Carlsbad, CA, USA). Viral RNA was isolated from cell-free supernatant using QIAmp Viral RNA mini kit (Qiagen, Germantown, MD, USA). Eluate was treated with RNase-free DNase I (Qiagen, Germantown, MD, USA) for 10 min at 37 °C, then heat-inactivated for 15 min at 70 °C. Following DNase treatment, RNA was purified using the Monarch RNA Cleanup kit (New England Biolabs (NEB), Ipswich, MA, USA). Purified RNA eluate was quantified by Nanodrop (Thermo Fisher Scientific, Waltham, MA, USA). Primers and probes were designed using Integrated DNA Technologies PrimerQuest tool (IDT) for RM housekeeping gene telomerase reverse transcriptase (tert) and SHIV.D pol, gag, and env, and were checked for specificity, dimers, and hairpins using MFEprimer 3.1 (iGeneTech, Beijing, China). Primer and probe sequences used for SHIV.D are as follows (Table S3). qPCR was performed using Luna Universal qPCR Master Mix (NEB, Ipswich, MA, USA) according to the manufacturer’s recommendations at the following cycling conditions: 1 min denaturation at 95 °C, 40 cycles of denaturation (15 s at 95 °C), and extension (30 s at 60 °C), followed by plate reading.

RT-qPCR was performed using Luna Universal One-Step RT-qPCR Kit (NEB, Ipswich, MA, USA) according to the manufacturer’s recommendations at the following cycling conditions: 10 min reverse transcription at 55 °C, 1 min denaturation at 95 °C, 40 cycles of denaturation (10 s at 95 °C), and extension (30 s at 60 °C), followed by plate reading. qPCR and RT-qPCR reactions were run on a Roche LightCycler 96 and analyzed on LC96 v. 1.1.0.1320 (Roche, Basel, Switzerland).

### 4.4. MDM EV Isolation

EVs were isolated from the cell culture supernatant of primary myeloid cells in vitro. Supernatant was collected and centrifuged as described above. Cell free supernatant was incubated with ExoQuick-TC (Systems Biosciences, Palo Alto, CA, USA) at a ratio of 5 mL supernatant: 1 mL ExoQuick overnight at 4 °C. Following incubation, EV/ExoQuick mixtures were centrifuged for 10 min at 3000× *g* to pellet EVs for downstream applications. Centrifugation was repeated if necessary to remove residual ExoQuick and cell culture media.

### 4.5. ZetaView Nanoparticle Tracking Analysis (NTA)

Isolated EV size distribution and concentration were measured using ZetaView nanoparticle tracker and analysis software (version 8.05.12 SP2; Particle Metrix, Ammersee, Germany). The instrument was calibrated before analysis using 100 nm polystyrene standard particles (Life Technologies, Carlsbad, CA, USA) for autoalignment and cleaned with sterile PBS and distilled water (dH_2_O). EV suspensions were diluted to optimal NTA concentration (50–300 particles/frame) in sterile PBS, and 2 mL of each sample was injected into the instrument for recording. Between each sample recording, the instrument was flushed with PBS until <5 particles were detected. NTA video was captured at sensitivity = 85, shutter speed = 100, and frame rate = 30 f/s. Twelve positions were traced for analysis. To account for increased cell death and decreased proliferation in the SHIV.D-infected cells in vitro, total EV concentration/mL was normalized for all samples by dividing EV concentration/mL by the qPCR endpoint fluorescence of housekeeping gene RM tert cellular DNA [54].

### 4.6. EV Infectivity Assay

Ghost(3) (G(3)) reporter cells, which express GFP upon HIV/SIV-Tat activation of transcription, were cultured for 5 days in selection media (DMEM (Thermo Fisher Scientific, Waltham, MA, USA) 10% FBS (Thermo Fisher Scientific, Waltham, MA, USA) with puromycin, Hygromycin B (Invitrogen, Carlsbad, CA, USA), and G418 geneticin (Thermo Fisher Scientific, Waltham, MA, USA) and maintained in DMEM supplemented with 10% FBS (Thermo Fisher Scientific, Waltham, MA, USA) and gentamicin (Corning, Corning, NY, USA) [68]. Primary MDMs were differentiated and infected as previously described. EVs were isolated from uninfected MDM and SHIV+ MDM and counted using ZetaView NTA. EVs suspended in sterile PBS were added to plated G(3) cells in maintenance media at a concentration of 500 EVs/cell in triplicate. As a positive control, G(3) cells were infected with SHIV.D at an MOI of 0.5 in triplicate. After 24 h, G(3) cells were collected for flow cytometry and DNA lysis. For GFP flow cytometry, cells were pelleted and resuspended in PBS with 2% PFA. GFP+ cells were counted using Guava easyCyte (Agilent Technologies, Santa Clara, CA, USA). Cell pellets were lysed for SHIV DNA qPCR, and DNA extraction was performed using a DNA miniprep kit (Zymo Research, Irvine, CA, USA). Double-stranded DNA was quantified using Qubit dsDNA BR Assay (Invitrogen, Carlsbad, CA, USA). qPCR was performed and analyzed as described above.

### 4.7. EV Marker Detection Arrays

Pelleted EVs were lysed using Exo-Check Exosome Antibody Array lysis buffer (Systems Biosciences, Palo Atlo, CA, USA). Following lysis, protein concentration was measured on a BioTek Cytation 1 microplate reader (Invitrogen, Carlsbad, CA, USA) using Pierce 660 nm Protein Assay Reagent (Thermo Fisher Scientific, Waltham, MA, USA). A total of 50 µg protein lysate was incubated with Exo-Check Exosome Antibody Arrays (Systems Biosciences, Palo Alto, CA, USA) according to the manufacturer’s instructions. Array containing antibodies against EV markers CD81, CD63, epithelial cellular adhesion molecule (EpCAM), annexin A5 (ANXA5), TSG101, FLOT1, intercellular adhesion molecule-1 (ICAM1), and ALIX were developed with SuperSignal™ West Femto Maximum Sensitivity Substrate (Thermo Fisher Scientific, Waltham, MA, USA) for 5 min at room temperature and imaged using an iBright FL1000 imaging system (Invitrogen, Carlsbad, CA, USA). Golgi matrix protein GM130 was included as a negative control for cellular contaminants.

### 4.8. Rhesus Macaques

RMs were maintained at Bioqual, Inc. (Rockville, MD, USA) according to the Association for Assessment and Accreditation of Laboratory Animal Care standards and all experiments were approved by the Bioqual, Inc. (Rockville, MD, USA). Institutional Animal Care and Use Committee. RMs were sedated with ketamine HCl at 10 mg/kg body weight for blood draws and SHIV inoculation. Peripheral blood was drawn into EDTA anticoagulant-containing tubes (purple top). RMs were intravenously infected with high titer SHIV.D.191859 challenge stock. After plasma viral load (VL) set point was established, daily subcutaneous combination ART (TDF 5.1 mg/kg, FTC 40 mg/kg, and DTG 2.5 mg/kg) administration began 10 weeks post infection. Blood was collected before infection and at several points over the course of acute infection and ART-mediated viral suppression (Figure 2A). Plasma viral loads were quantified using RT-qPCR by the Nonhuman Primate Virology Core Laboratory at Duke University (Durham, NC, USA) (Figure 2B).

### 4.9. Plasma EV Isolation

Blood samples were gently inverted 6–8 times, then centrifuged at 2000 rpm for 20 min at room temperature (RT) to separate the plasma layer. Plasma was aliquoted into microcentrifuge tubes (1 mL/tube) and centrifuged at 17,000× *g* for 5 min to remove platelets and cellular debris (Figure S1). Platelet-poor plasma samples were transferred to new microcentrifuge tubes and stored at −80 °C. Plasma from each RM (n = 10) at three timepoints (pre-infection, during acute viremia at 11 days post-infection (DPI) and during ART-mediated viral suppression at 167 DPI) were used for analysis. Platelet-free plasma was incubated with Thrombin (Systems Biosciences, Palo Alto, CA, USA) at a ratio of 500 µL plasma:4 µL Thrombin for 5 min at room temperature and centrifuged at 10,000 rpm for 5 min to remove fibrin and fibrinogen. EVs were precipitated from 250 µL fibrin-free plasma by incubation with 63 µL ExoQuick (Systems Biosciences, Palo Alto, CA, USA) for 30 min at 4 °C, followed by centrifugation at 1500× *g* for 30 min at 4 °C. Pelleted EVs were resuspended in 400 µL sterile phosphate-buffered saline (PBS; Corning, Corning, NY, USA) and filtered through 0.22 µm microcentrifuge tube fitted filters (Millipore Sigma, Burlington, MA, USA) by centrifugation at 1000× *g* for 10 min at RT. Purified EV preparations at this step were used for characterization by ZetaView nanoparticle tracking analysis (NTA) and EV marker antibody array.

### 4.10. Flow Cytometry Antibodies and Targets

The goal of our research was to develop a method to quantify EVs from different cell types of origin in plasma. We used one common tetraspanin marker for plasma EVs (CD81) and specific markers for six cell subsets (macrophages, lymphocytes, neurons, microglia, monocytes, and endothelial cells) to determine EV origin (Table S1). The anti-human monoclonal antibodies used in this study were CD11b-BUV 737 (clone ICRF44, Thermo Fisher Scientific, Waltham, MA, USA), CD14-Pacific Blue (clone M5E2) and CD31-BUV 395 (clone WM59) (BD Biosciences, Franklin Lakes, NJ, USA), CD171/L1CAM-AlexaFluor 488 (clone 2702C, Novus Biologicals, Centennial, CO, USA), CD3-APC-Cy7 (clone OKT3) and CD81-PE-Cy7 (clone 5A6) (Biolegend, San Diego, CA, USA), and TMEM119-AlexaFluor 700 (clone 1023426, R&D Systems, Minneapolis, MN, USA) (Table S1). Marker clones were chosen for known NHP reactivity using the Non-human Primate Reagent Resource reactivity database (NHPRR).

### 4.11. EV Flow Cytometry and Instrumentation

A BD FACSymphony A5 Spectral Enabled (SE) flow cytometer (BD Biosciences, Franklin Lakes, NJ, USA) was used to conduct the analysis. The instrument is equipped with 5 lasers: blue (488 nm excitation, 150 mW power), red (637 nm, 140 mW), violet (405 nm, 200 mW), yellow-green (561 nm, 150 mW), and ultraviolet (355 nm, 60 mW), as well as 28 filters configured as 6B-3R-8V8UV-5YG, and square photomultiplier tubes (PMT) optimized for cascade array. Filtered EV suspensions prepared as previously described were incubated with 5 µL human Fc seroblock (BioRad, Hercules, CA, USA) for 10 min at RT, transferred to sterile round-bottom polystyrene tubes (Corning, Corning, NY, USA), then stained with antibodies for 30 min at 4 °C protected from light. Following antibody incubation, 400 µL EV suspensions were fixed with 125 µL 4% paraformaldehyde (PFA) in PBS for a final concentration of 1% PFA. With each set of samples, a sterile PBS only control, negative EV control, pre-blocking EV control, sterile PBS 1% PFA with antibody cocktail control, single stain controls, and fluorescence minus one (FMO) controls were run in accordance with recommendations made by the MIFlowCyt-EV reporting framework [46,69]. A membrane disruption control of unstained EVs lysed with 1% Triton X-100 (Thermo Fisher Scientific, Waltham, MA, USA) for one hour was also used [48].

To ensure data resolution and avoid background noise, BD FACSymphony A5 SE (BD Biosciences, Franklin Lakes, NJ, USA) was put through vigorous cleaning and system preparation. For each session, a specialized BD Detergent Solution (BD Biosciences, Franklin Lakes, NJ, USA) was run through the instrument for 15 min, followed by 15 min of autoclaved deionized water. The sheath fluid in the system was thoroughly replaced with autoclaved deionized water obtained from the Millipore Milli-Q Direct 16 water purification system (Millpore, Burlington, MA, USA) for one hour. A PureFlo^®^ Capsule filter (ZenPure Americas, Manassas, VA, USA) was added to the fluidics line. Autoclaved deionized water was run through the instrument for one hour to minimize bubble formation. Daily calibration was conducted with BD Cytometer Set-up and Tracking beads. The cytometer detection threshold was optimized and set to 250. This cleaning and calibration procedure was performed for every data acquisition run, and the instrument was flushed by a sterile PBS run between controls and samples. Data, including mean fluorescence intensity (MFI) of EV sub-populations, were calculated using FlowJo_v10.9.0 (TreeStar, Inc., Ashland, OR, USA). Graphics were generated by FlowJo or created in BioRender.com.

### 4.12. In-Solution Digestion for Proteomics

Primary MDM were differentiated from the PBMC of 6 healthy RMs, and half of the MDMs from each RM were infected with SHIV.D as described above. A total of 10 mL of pooled cell culture supernatant from DPI 2–8 was harvested at the same time from SHIV.D−/+ MDM and used for EV isolation and proteomic analysis. EV pellets underwent lysis, solubilization, and digestion on an S-Trap (Protifi, Fairport, NY, USA) following the manufacturer’s protocol [70]. Subsequently, the resulting proteins were de-salted using an Oasis HLB plate (Waters, Milford, MA, USA), dried via vacuum centrifugation, and reconstituted in 0.1% TFA containing iRT proteins (Biognosys Schlieren, Schlieren, Switzerland). An extended description of proteomics methods is provided in the Supplementary Data.

### 4.13. Mass Spectrometry Acquisition and Data Analysis

Proteins were analyzed on an Exploris 480 mass spectrometer (Thermo Fisher Scientific, San Jose, CA, USA) coupled with an Ultimate 3000 nano UPLC system (Thermo Fisher Scientific, Waltham, MA, USA) and an EasySpray source utilizing data independent acquisition (DIA) (Thermo Fisher Scientific, Waltham, MA, USA). Subsequently, raw data were searched using Spectronaut version 19, and the bioinformatics analysis was conducted in R version 4.3.3.

### 4.14. Statistics and Analysis

In analysis, proteins that were detected in at least 4 of the 6 replicates of at least one condition were included. Comparisons and statistical analyses were performed using GraphPad Prism version 9.4.1 (La Jolla, CA, USA). For MEV NTA data set, the N value was insufficient to determine a normal distribution by the D’Agostino–Pearson test. Therefore, nonparametric Wilcoxon matched-pairs signed rank tests were used to compare groups. Descriptive statistics were also calculated for each MEV group. NTA graphs display individual values. In bar graphs representing PCR and flow cytometry data, the bar represents the mean value of technical replicates. Error bars represent the standard error of the mean. To analyze PEV NTA data, results were first tested for normal (Gaussian) distribution using the D’Agostino–Pearson test (*p* < 0.05) (GraphPad Prism version 9.4.1, La Jolla, CA, USA). All PEV NTA data sets assumed normal distribution. Descriptive statistics were calculated for each group, and parametric paired *t*-tests were run to compare groups. To compare EV sub-population MFI between groups, all experimental data sets were again tested for normality using the D’Agostino–Pearson test. Of the six parameters texted, two assumed a Gaussian distribution (CD171, TMEM119), and four did not (CD11b, CD14, CD3, CD31). In data sets that did assume a normal distribution, repeated measures one-way ANOVA were run, followed by post hoc paired *t*-tests (Dunnett’s multiple comparisons tests) compared to the pre-infection control group. In data sets that were not distributed normally, Friedman one-way repeated measure analysis of variance tests were run, followed by Dunn’s multiple comparisons tests. All graphs display individual values with no exclusion of outliers. In bar graphs, the bar represents the mean value with error bars representing the standard error of the mean. Proteomic bioinformatics analysis was conducted in R version 4.3.3.

## Data Availability

The original contributions presented in this study are included in the article and Supplementary Materials. Further inquiries can be directed to the corresponding author.

## Supplementary Materials

The following supporting information can be downloaded at https://www.mdpi.com/article/10.3390/ijms27020966/s1.
