# Supplementary material for: SHIV.D Infection Alters Production and Protein Composition of Myeloid-Derived Extracellular Vesicles
Source: Int J Mol Sci. 2026 Jan 18;27(2):966. doi: 10.3390/ijms27020966 (PMC12842161; doi:10.3390/ijms27020966)
Supplement: Supplementary file 1 [file ijms-27-00966-s001.zip › ijms-4077482-supplementary.pdf]

Supplementary Material

Rachel M. Podgorski[1], Amir Yarmahmoodi[2], Stephen Baak[1], Rebecca Warfield[1], Jake A. Robinson[3], Jennifer Roof[4], Hossein Fazelinia[4], Maurizio Caocci[5], Lynn A. Spruce[4], Katharine J. Bar[3] and Tricia H. Burdo[5*]

[1]Center for NeuroVirology and Gene Editing, Department of Microbiology, Immunology, and Inflammation, Lewis Katz School of Medicine, Temple University, Philadelphia, PA, USA.

[2]Flow Cytometry Core Facility, Department of Microbiology, Immunology, and Inflammation, Lewis Katz School of Medicine, Temple University, Philadelphia, PA, USA.

[3]Department of Medicine, Perelman School of Medicine, University of Pennsylvania, Philadelphia, PA, USA.

Proteomics Core Facility, Children's Hospital of Pennsylvania and the University of Pennsylvania, Philadelphia, PA, USA.

[5]Department of Medicine, Institute of Translational Medicine and Science, Robert Wood Johnson Medical School, Rutgers, The State University of New Jersey, New Brunswick, NJ, USA.

*Corresponding Author: Tricia H. Burdo, Ph.D., tb874@rbhs.rutgers.edu

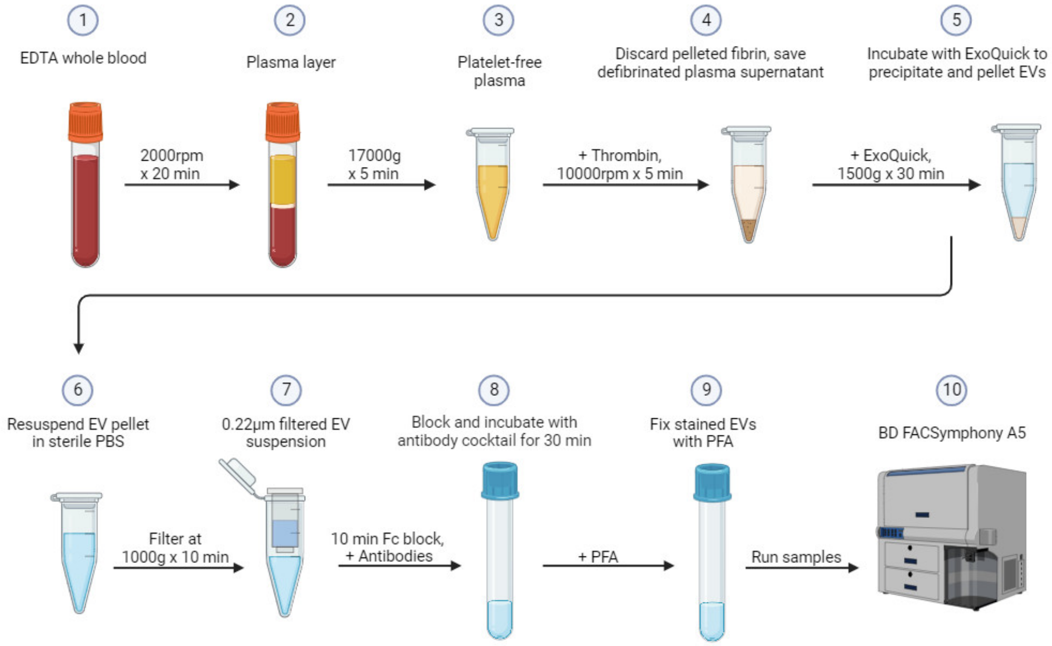

Figure S1. PEV flow cytometry workflow. 10mL EDTA anticoagulant-treated whole blood samples are centrifuged at 2000rpm for 20 minutes at room temperature to separate the plasma layer. 1mL aliquots of cell-free plasma is transferred into microcentrifuge tubes and centrifuged at 17000g for 5 minutes at room temperature (RT) to obtain cell debris-free, platelet-free plasma. For each sample, 335µL plasma is incubated with 2.68µL Thrombin (SBI) for 5 minutes at RT, then centrifuged at 10000rpm for 5 minutes at RT to pellet fibrin. 250µL fibrin-free plasma is transferred to a fresh microcentrifuge tube and incubated with 67µL ExoQuick (SBI) for 30 minutes at 4°C to precipitate EVs. After incubation, the plasma/ExoQuick mixture is centrifuged at 1500g for 30 minutes at 4°C to pellet EVs. The EV pellet is resuspended in 400µL sterile PBS and pipetted into a 0.22µm (220nm) filter chamber fitted in a microcentrifuge collection tube. The EV suspension is filtered by centrifugation at 1000g for 10 minutes at RT. Following centrifugation, the filters are discarded and 5µL Fc blocking solution is added to the EV suspension and incubated for 10 minutes at RT. After blocking, the EV suspension is transferred to a 5mL round-bottom polystyrene tube. The appropriate antibody cocktails are added to each sample and incubated for 30 minutes at 4°C, protected from light. 125µL 4% paraformaldehyde (PFA) in PBS is added to each sample for fixation to reach a total PFA concentration of 1%. Fixed, stained EV samples are analyzed on a BD FACSymphony A5 flow cytometer.

Antibody	Panel	Target	Amount per test	Fluoro-chrome	Supplier	Catalog #	Clone
CD11b	A	macrophage/myeloid-EVs	5μL	BUV 737	Thermo	367-0118-42	ICRF44
CD14	A	monocyte-EVs	2.5μL	Pacific Blue	BD	558121	M5E2
CD171	B	neuron-EVs	5μL	AlexaFluor 488	Novus	FAB7773G	2702C
CD3	B	T lymphocyte-EVs	5μL	APC-Cy7	Biolegend	317342	OKT3
CD31	B	endothelium-EVs	5μL	BUV 395	BD	565290	WM59
CD81	A&B	EVs (tetraspanin)	5μL	PE-Cy7	Biolegend	349512	5A6
TMEM119	A	microglia-EVs	5μL	AlexaFluor 700	R&D	FAB10313N	1023426

Table S1. Flow cytometry antibodies used, their targets, panels, and specifications. Abbreviations: transmembrane protein 119 (TMEM119), allophycocyanin (APC), brilliant ultraviolet (BUV), cyanine 7 (Cy7), phycoerythrin (PE), BD Biosciences (BD), R&D Systems (R&D).

**Panel A**					**Panel B**				
	AlexaFluor 700	BUV737	Pacific Blue	PE-Cy7		APC-Cy7	AlexaFluor 488	BUV395	PE-Cy7
AlexaFluor 700	X	12.881	1.009	0.716	APC-Cy7	X	0.089	0.052	33.244
BUV737	12.933	X	0.3053	0.085	AlexaFluor 488	0	X	1.89	0
Pacific Blue	0	0.172	X	0	BUV395	0	0.265	X	0
PE-Cy7	0.228	10.61	-0.4	X	PE-Cy7	12.218	13.392	20.305	X

Table S2. Compensation matrices of panels A and B. Compensation matrices were obtained using single color compensation EV sample controls for each parameter. Spectral overlap between fluorochromes was calculated using FlowJo 10.9.0. Abbreviations: allophycocyanin (APC), brilliant ultraviolet (BUV), cyanine 7 (Cy7), phycoerythrin (PE).

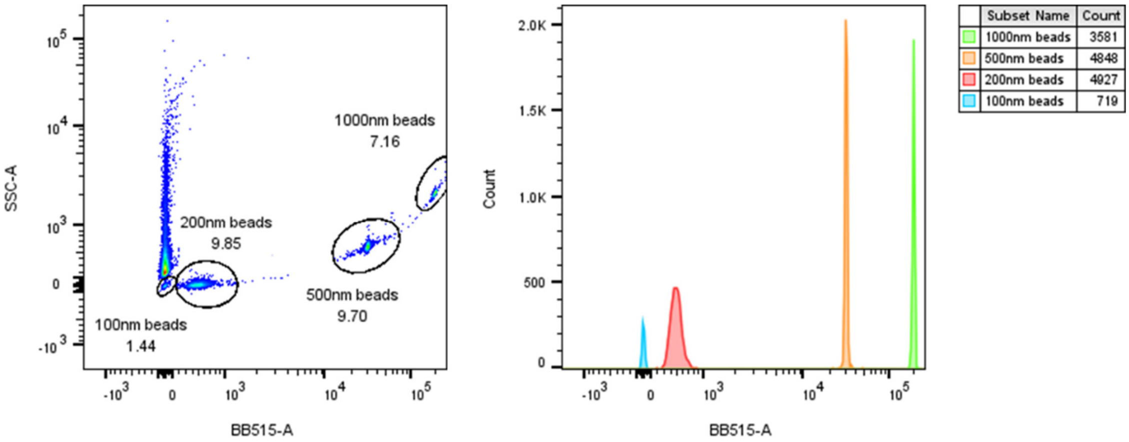

Figure S2. Sub-micron reference bead detection and SSC. 100nm, 200nm, 500nm, and 1000nm particle size reference bead populations were located by fluorescence intensity on the BB515 filter and side scatter area (SSC). Reference bead populations are shown in elliptical gates with the percentage of total events in each population (left). Total event counts by fluorescence intensity of each bead population are shown as 100nm: blue, 200nm: red, 500nm: orange, 1000nm: green (right). Event count peaks by fluorescence intensity and total subpopulation counts were calculated using FlowJo 10.9.0.

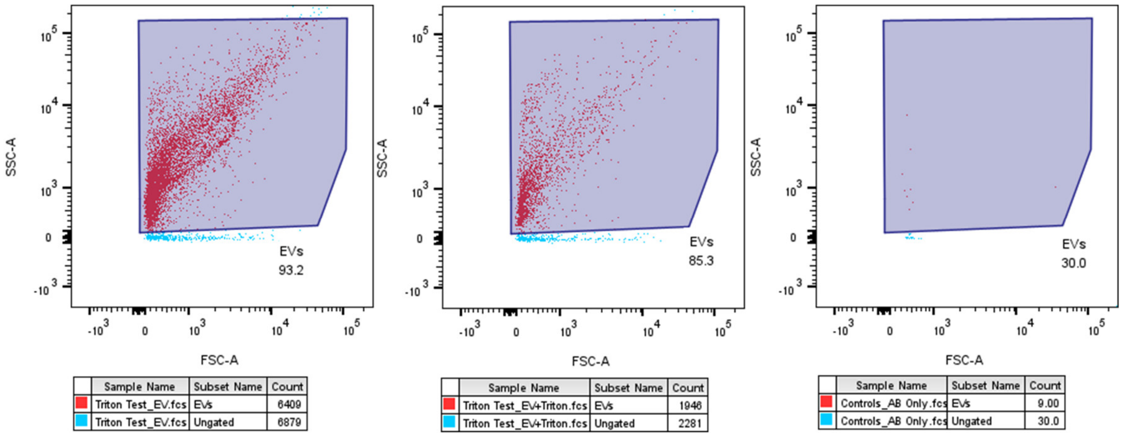

Figure S3. SSC/FSC gating on unstained EV control, lysed control, and antibody only control. An unstained EV control sample was used to determine baseline side scatter area/forward scatter area (SSC/FSC) gating (left). An unstained EV control treated with 1% Triton X-100 detergent to lyse EV membranes was recorded (center) and demonstrated a 70% reduction in EV events. A control sample containing only the antibody cocktail (Panel A + Panel B antibodies) suspended in sterile PBS 1% PFA was used to ensure antibody aggregates were not being recoded as false positive events (right). Events with appropriate EV SSC/FSC fall inside “EVs” subset gate (blue shading) and appear as red dots. Events outside “EVs” subset gate appear as blue dots and were excluded from compensation and analysis. Percentage of events falling inside EV gate is denoted in bottom right corners. Event counts, gating, and figure generation was performed using FlowJo 10.9.0.

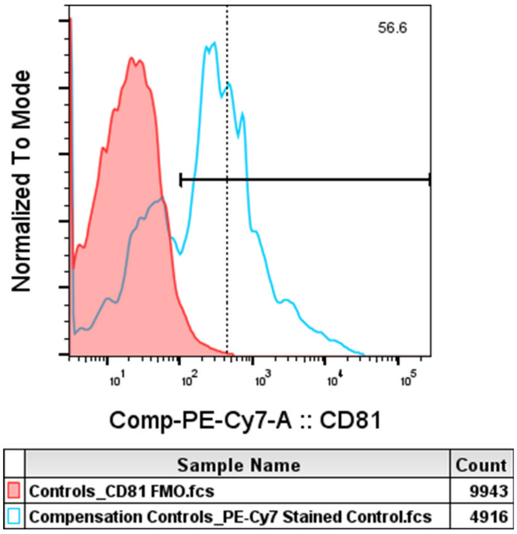

Figure S4. CD81+ EV gating. After setting EV side scatter area (SSC) and forward scatter area (FSC) gates, CD81+ positive signal gating (black horizontal line) was determined using single color compensation controls (blue outline) and FMO controls (red shading) in FlowJo 10.9.0. Percentage of CD81+ positive events in the compensation control sample is denoted in the top right corner (56.6% CD81+). Mean fluorescence intensity (MFI) of the positive subpopulation is represented by a vertical black dotted line.

Target	Forward primer	Reverse primer	Probe
RM-*tert*	5'-GAG CTG AGA TTG TGC CCT TG-3'	5'-CCA TTT GCT CTC TGC TC-3'	5'-/5HEX/CCA GCA CAG ATC CTG GTC CCG T/3BHQ-1/-3'
SHIV-*pol*	5'-CAC TTA CCA GTT GAG AGG GAT G-3'	5'-CTT ACT AGT GGT GGT GTT GAG ATA A-3'	5'-/56-FAM/ACC TGC CAA /ZEN/TAG TCT GTC CAC CAC /3IABkFQ/-3'
SHIV-*gag*	5'-GAA AGC CTG TTG GAG AAC AAA G-3'	5'-CCA GAT GAC GCA GAC AGT ATT A-3'	5'-/56-FAM/TAG CTC CAT /ZEN/TAG TGC CAA CAG GCT /3IABkFQ/-3'
SHIV-*env*	5'-CCA CCA AGG CAA AGA GAA GA-3'	5'-CTT CCT GCT GTT CCC AAG AA-3'	5'-/56-FAM/AGA GCA ATA /ZEN/GGA CTA GGA GCC CTG T/3IABkFQ/-3'

Table S3. Primers and probes for SHIV.D/RM qPCR and RT-qPCR.

Gene	logFC	AvgExpr	t	P value	adj.P value	B
**LCN2**	3.655438	-0.547926	9.4125355	0.0000205	0.0285549	3.287348
**PLEKHB2**	-3.190891	-0.8254686	-8.730479	0.0000346	0.0285549	2.8399919
**MMP7**	-3.363916	-0.2085002	-8.535004	0.0000404	0.0285549	2.704196
**COCH**	-2.635825	0.6983779	-7.866633	0.0000704	0.0307947	2.2102459
**LDLR**	-1.95001	2.0987059	-7.831565	0.0000726	0.0307947	2.183
**SNRPA**	-2.572535	-2.1176433	-7.47828	0.0000991	0.0327808	1.9006804
**CD36**	-2.476447	-0.4186557	-7.380335	0.0001082	0.0327808	1.8198191
**CD63**	-1.846775	2.2530424	-6.9303935	0.0001641	0.0404983	1.4336796
**PRCP**	-1.624058	0.0601066	-6.845243	0.0001781	0.0404983	1.3571346
**COLEC12**	-2.452713	0.7369188	-6.763647	0.0001927	0.0404983	1.2833531
**RALA**	-1.70828	-2.9713985	-6.675185	0.00021	0.0404983	1.2023623
**ITGAX**	-1.472674	2.3712421	-6.535937	0.0002409	0.0425833	1.0727348
**ARRDC1**	-1.769798	1.0675878	-6.249813	0.0003216	0.0471465	0.7979477
**HTRA1**	-1.348342	3.6647814	-6.157305	0.0003538	0.0471465	0.706618
**ITGB2**	-1.47168	3.6831326	-6.01934	0.0004087	0.0471465	0.5680996
**HMGN3**	3.866067	-2.0630072	5.949581	0.00044	0.0471465	0.4969932
**WDR83OS**	-4.266097	-0.9657041	-5.871243	0.0004783	0.0471465	0.4162768
**LPL**	-2.151474	5.357892	-5.823097	0.0005037	0.0471465	0.3662111
**CD53**	-1.401456	1.8475491	-5.814905	0.0005082	0.0471465	0.3576577
**MRC1**	-2.39218	4.6909445	-5.794167	0.0005197	0.0471465	0.3359531

Table S4: Significantly differently abundant proteins between conditions. Gene: UniProt GeneID, logFC: log2 fold-change, t: t-statistic, P value: raw P value, adj.P value: adjusted P value (q-value), B: log-odds that the gene is differentially expressed.

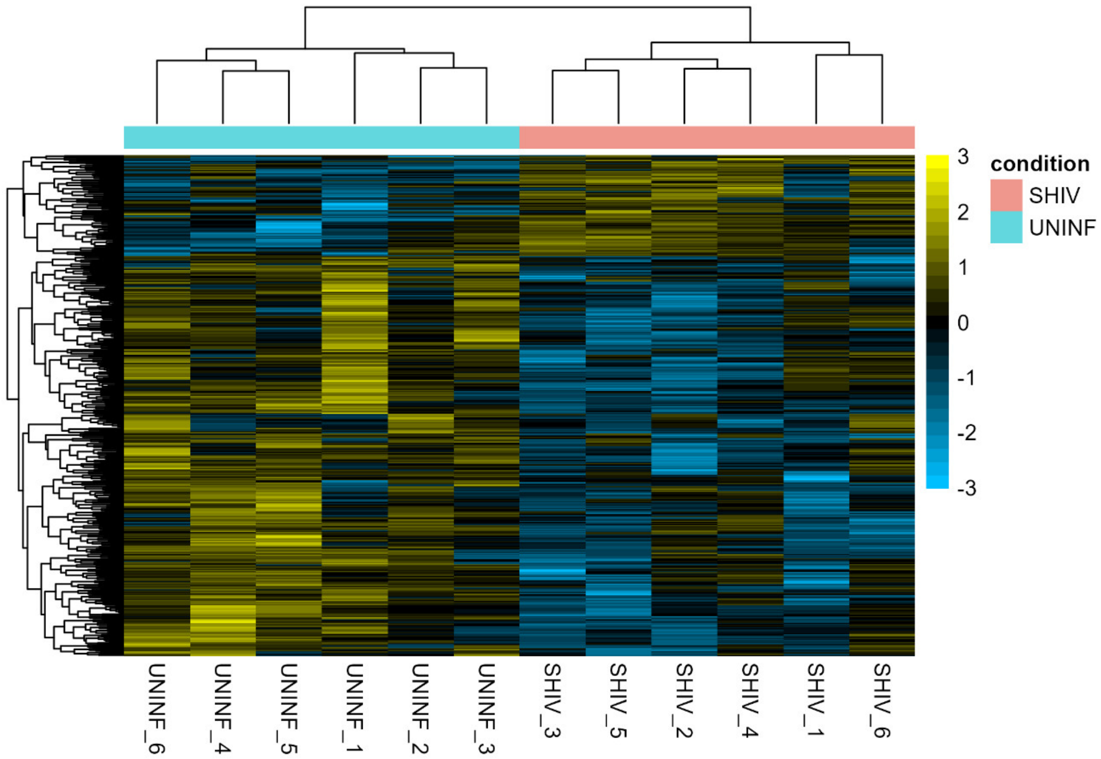

Figure S5. Heatmap of total significant proteins. An annotated heatmap clusters samples by fold change of significant proteins. The heatmap generated demonstrates clustering by condition.

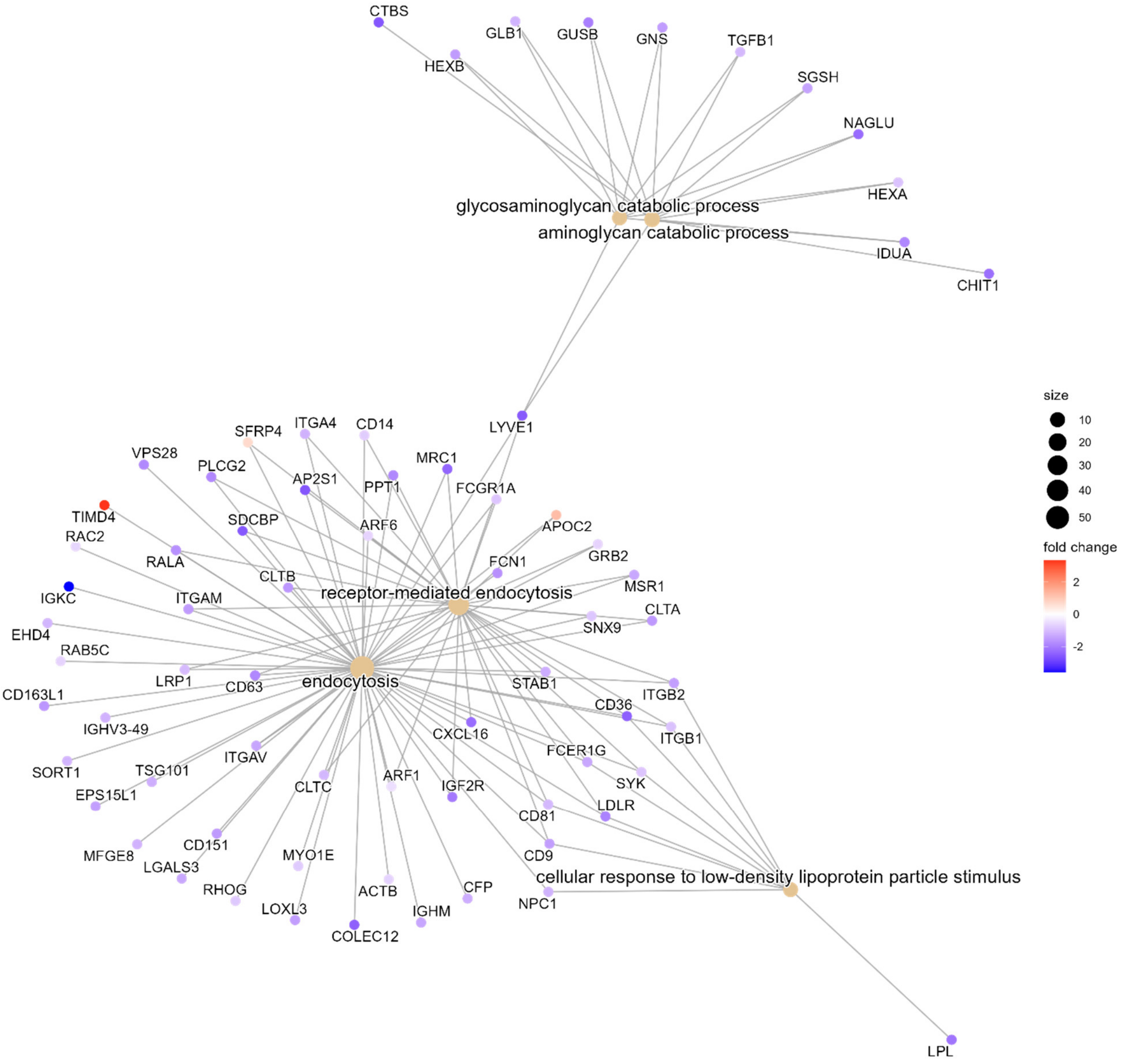

Figure S6. Enrichment map network of overrepresented GO pathway proteins. GO pathways with the largest number of associated proteins were plotted in a map network. Fold change of proteins associated with each GO term (SHIV vs. UNINF) is indicated by dot color. Blue: negative fold change, red: positive fold change.

Extended proteomics methods

Protein Extraction for Mass Spectrometry

Protein extraction for mass spectrometry (MS), MS, and subsequent bioinformatics analysis was performed by the University of Pennsylvania/Children's Hospital of Philadelphia Proteomics Core (Philadelphia, PA). EV pellets were solubilized in 50µL of extraction buffer containing 5% sodium dodecyl sulfate (SDS, Affymetrix), 50mM TEAB (pH 8.5, Sigma), and protease inhibitor cocktail (Roche complete, EDTA free). To shear DNA and ensure complete solubilization, samples were sonicated for 10 minutes at 20°C in a Covaris R230 focused-ultrasonicator with the following settings: Dithering: Y=3.0, Speed=20.0, peak incidence power: 360.0, duty factor: 30, cycles per burst: 200. Samples were centrifuged at 3000g for 10 minutes to clarify lysate. Protein concentration was determined by intrinsic tryptophan fluorescence, excited at 280nm and read at 350nm, against an in-house generated E. coli lysate standard curve on a Synergy H1 microplate reader (BioTek).

In-Solution Digestion

10µg of each sample was digested per the S-Trap Micro (Protifi) manufacturer's protocol. Briefly, proteins were reduced in 5mM TCEP (Thermo), alkylated in 20mM iodoacetamide (Sigma), then acidified with phosphoric acid (Aldrich) to a final concentration of 1.2%. Samples were diluted with 90% methanol (Fisher) in 100 mM TEAB, then loaded onto an S-trap column and washed three times with 90% methanol in 100 mM TEAB. A 1:10 ratio (enzyme: protein) of Trypsin (Promega) and LysC (Wako) suspended in 20µL 50mM TEAB was added, and samples were digested for 18 hours at 37°C in a humidity chamber. After incubation, peptides were eluted with an additional 40µL of 50 mM TEAB, followed by 40µL of 0.1% trifluoroacetic acid (TFA) (Pierce) in water, and finally 40µL of 50/50 acetonitrile: water (Fisher) in 0.1% TFA. Eluates were combined and organic solvent was dried off via vacuum centrifugation. Samples were then desalted using an Oasis HLB µElution plate (30um, Waters). Wells were conditioned two times with 200µL of acetonitrile and equilibrated three times with 200µL of 0.1% TFA. Samples were applied, washed three times with 200µL 0.1% TFA, and eluted directly into autosampler vials in three increments of 65µL of 50:50 acetonitrile: water. Eluates were then dried by vacuum centrifugation and reconstituted in 0.1% TFA containing iRT peptides (Biognosys, Schlieren, Switzerland). Peptide concentration was determined at OD280 using a Synergy H1 microplate reader (BioTek), and samples were adjusted to 400ng/µL for injection.

Mass Spectrometry Data Acquisition

Samples were randomized and analyzed on an Exploris 480 mass spectrometer (Thermo Fisher Scientific San Jose, CA) coupled with an Ultimate 3000 nano UPLC system and an EasySpray source. 5µl of sample was loaded onto an Acclaim PepMap 100 75µm x 2cm trap column (Thermo) at 5uL/min and separated by reverse phase (RP)-HPLC on a nanocapillary column, 75µm id × 50cm 2um PepMap RSLC C18 column (Thermo). Mobile phase A consisted of 0.1% formic acid and mobile phase B of 0.1% formic acid/acetonitrile. Peptides were eluted into the mass spectrometer at 300nL/min with each RP-LC run comprising a 105-minute gradient from 3% B to 45% B.

Data independent acquisition (DIA) mass spectrometer settings were as follows: one full MS scan at 120,000 resolution, with a scan range of 350-1200 m/z and normalized automatic gain control (AGC) target of 300%, and automatic maximum inject time. This was followed by variable (DIA) isolation windows, MS2 scans at 30,000 resolution, a normalized AGC target of 1000%, and automatic injection time. The default charge state was 3, the first mass was fixed at 250 m/z, and the normalized collision energy for each window was set at 27.

Quality Analysis/Quality Control (QA/QC) and System Suitability

The suitability of Exploris 480 instrument was monitored using QuiC software (Biognosys; Schlieren, Switzerland) for the analysis of the spiked-in iRT peptides. As a measure for quality control, we injected standard E. coli protein digest in between samples (one injection after every four biological samples) and collected the data in data dependent acquisition (DDA) mode. The collected DDA data were analyzed in MaxQuant (200) and the output was subsequently visualized using the PTXQC package to track the quality of the instrumentation.

Database Searching

The DIA raw files were processed using Spectronaut 18.7 in direct DIA mode. We utilized a RM database comprising canonical and reviewed isoforms from UniProt, supplemented with a list of 245 common protein contaminants and iRT peptides. Enzyme specificity was set to trypsin with allowance for two potential missed cleavages. Fixed modification was specified as carbamidomethyl of cysteine, while protein N-terminal acetylation and oxidation of methionine were considered variable modifications. To ensure high confidence, a false discovery rate limit of 1% was applied for precursors, peptides, and proteins identification, while the remaining search parameters were maintained at their default settings.

Bioinformatics Analysis for Proteomics

Proteomics data processing and statistical analysis were conducted in R. The MS2 intensity values generated by Spectronaut were utilized for analyzing the entire proteome dataset. The data underwent log2 transformation and normalization by subtracting the median value for each sample. To ensure data integrity, we filtered it to retain only proteins with complete values in at least one cohort. To compare proteomics data across groups, we employed a Limma paired t-test to identify proteins with differential abundance, and we visualized the impact of these differences through volcano plots. Lists of differentially abundant proteins were generated based on criteria of P value <0.05, resulting in a prioritized list for subsequent bioinformatics analysis.
